# Poison of Putrefied Blood

**Published:** 1878-01

**Authors:** 


### Poison of Putrefied Blood.

M. Feltz, to whose researches on the "intimate pathology" we have before referred, has communicated to the Académie des Sciences a fresh series of experiments instituted with the object of confirming his previous observations, and of proving that putrefied, toxic blood contains no liquid or solid virus except the organised ferments.

Thirty grammes of very poisonous blood, mixed with 100 cubic centimetres of distilled water, were subjected to a heat of 80° cent. for ten minutes, so as to solidify all the coagulable constituents. The liquid portion, separated by filtering, was rich in vibrios. It was then evaporated *in vacuo* to fifty cubic centimetres, and five cubic centimetres injected into two rabbits killed them in two or three days with all the symptoms of septicæmia. The same liquid was then filtered through a layer of cotton and charcoal; the filtrate was a clear rose-colored liquid, which contained neither bacteria, vibrios, nor moving granules. Six cubic centimetres of this solution were injected into the jugular vein of each of four rabbits without the slightest effect. M. Feltz assumes from this that the existence of a soluble ferment of liquid virus is highly improbable. He does not, however, allude to the possible influence of the charcoal through which the liquid was filtered, and which may have destroyed the specific properties of a liquid virus, had such existed.

At the suggestion of Claude Bernard, he has made further observations on the degree in which the organised particles obey the law of gravitation, and, as has been asserted in the case of the virus of vaccinia and glanders, fall to the lowest strata of the liquids which hold it in suspension, and do not float in all parts of the liquid, as do cryptomagic ferments. Four or five cubic centimetres of putrefied blood reduced to a syrupy consistence, and possessing highly toxic qualities, were placed in each of four upright glasses, and covered with a layer of distilled water five centimetres high. In a fifth glass some distilled water was placed, rendered slightly alkaline by an ammoniacal salt. The five glasses were then protected from movement or disturbance for four days. At the end of that time five cubic centimetres, taken from the surface of each glass, were injected into the five rabbits; all of which died in a few days, excepting the rabbit into which alkaline water had been injected. The surface of the liquid in the glasses in which the blood had been placed contained an enormous quantity of "organised ferments," while the alkaline water contained only some atmospheric dust. In another experiment two quantities of diluted toxic blood, prepared as above described, were placed in tall narrow test-tubes, and kept free from all disturbance, together with a third similar tube containing alkaline distilled water. Solid inorganic grains should sink to the bottom of the test-tube, and the superficial layers of the solution, if the inorganic granules are the vehicles of contagion (as M. Chauveau asserts with regard to vaccine virus), should be innocuous. But microscopical examination revealed abundant vibrios in the superficial layers of the dilution, and experiment showed that these superficial layers were as toxic as the deeper layers, since four rabbits inoculated from different layers died very rapidly of septicæmia, while one in which the superficial layer of the alkaline water was injected, remained unaffected

The conclusions drawn by M. Feltz are, that there are no infectious ferments of the nature of diastaste in toxic blood nor any liquid virus, nor any solid unorganised virus; that a heat of 150° Centigrade, which destroys all toxic quality, acts only on the organised ferments, the true septic agents.—*Lancet.*

